# Novel screw-cable integrated system(SCIS) for minimally invasive treatment of patella transverse fractures: a finite element analysis

**DOI:** 10.1186/s13018-023-04306-2

**Published:** 2023-10-31

**Authors:** Songyang Liu, Shen Liu, Feng Gu, Xing Wei, Yonghui Liang

**Affiliations:** Aerospace Center Hospital, Beijing, China

**Keywords:** Modified tension band wiring, SCIS, Patella transverse fractures, Finite element analysis

## Abstract

**Background:**

The most recommended method for treating transverse patella fractures is modified tension band wiring (MTBW). However, the optimal instrument for use with MTBW is still undetermined. Hence, we aimed to design a novel screw-cable integrated system (SCIS) and compare its biomechanical characteristics with Kirschner-wire, SCIS, and Cable-Pin systems in treating transverse patellar fracture.

**Methods:**

A finite-element (FE) model of transverse patella fracture was created. The fracture model was fixed with either *K*-wire, SCIS, or Cable-pin. Different tension force loading (400 N and 800 N), direction(0° and 45°), and screw or *K*-wire depth(5 mm and 10 mm) were set. The maximum displacement of the fragment and maximum gap opening were measured by using FE analysis.

**Results:**

Compared with the *K*-wire and Cable-pin system, SCIS increased the stability of the fractured patella by reducing fragment displacement and gap opening. Under 400 N loading in the direction 45°, SCIS with screw placing at 5-mm depth reduced the maximum fragment displacement (0.43 mm) by 49.62% and 26%, respectively, compared with the *K*-wire (0.22 mm) and Cable-pin (0. 22 mm) group. Meanwhile, the gap opening in SCIS (0.05 mm) was reduced by 83% and 59.8% (0.05 to 0.18) compared with the *K*-wire (0.30 mm) and Cable-pin (0.18 mm) group.

**Conclusion:**

SCIS demonstrated improved biomechanical stability for treating transverse patellar fractures compared to MTBW with Kirschner wire and the Cable-Pin system. Finite element analysis showed SCIS substantially reduced fracture fragment displacement and gap opening under various loading conditions.

## Introduction

Patella fractures account for approximately 1% of all skeletal injuries [1–3] and present a significant challenge for orthopedic surgeons due to their unique anatomical structure. Transverse fractures are the most prevalent type, which frequently leads to dysfunction of the knee extensor mechanism due to displacement of the fractured segments [4, 5].

Surgery is essential when the fracture gap is larger than 2–3 mm or there is joint incongruence. The most recommended surgery for treating transverse patella fractures is modified tension band wiring (MTBW) [6–8], which was recommended by AO Group. The optimal instrument for use with MTBW is still undetermined. Kirschner's wire (*K*-wire) was the earliest instrument to be used with MTBW. However, MTBW with *K*-wire has notable disadvantages including fixation loosening, higher infection risk, non-union and hardware irritation [9, 10]. Some researchers reported that screws provided stronger fixation than *K*-wire [11–13]. Therefore, TBW with various metal and non-metallic implants were developed, of which the most representative is the Cable-pin system [14–17]. Cable-pin system combines the advantages of screw fixation and TBW principle, allowing for more stable fixation and early mobilization of the knee joint. However, improper screw length can still irritate the quadriceps. Moreover, based on our clinical experience and the manufacturer's instruction, the Cable-pin system used solid screws that require repeated needle passes, and the compression strength still needs adjustment. There is also a lack of research on the biomechanics of patella fracture fixation instruments.

Given the limitations of existing studies of patellar fixation, it is of great importance to develop new instrument and surgical approaches. We develop the novel screw-cable integrated system (SCIS) by integrating the advantages of Cable-pin system, cannulated screws and TBW principle. With the unique design, the novel SCIS potentially offers enhanced stability and superior load-bearing capabilities. This study aims to provide a comprehensive biomechanical analysis of this new system and compare its performance to traditional treatment methods.

## Methods

In this study, we first designed and produced a novel SCIS system. Then, we developed a Finite Element (FE) model to simulate and compare the biomechanics of applying MTBW using SCIS with using *K*-wire or Cable-pin system for treating transverse patella fractures.

### The design and characteristics of SCIS

**Fig. 1:**
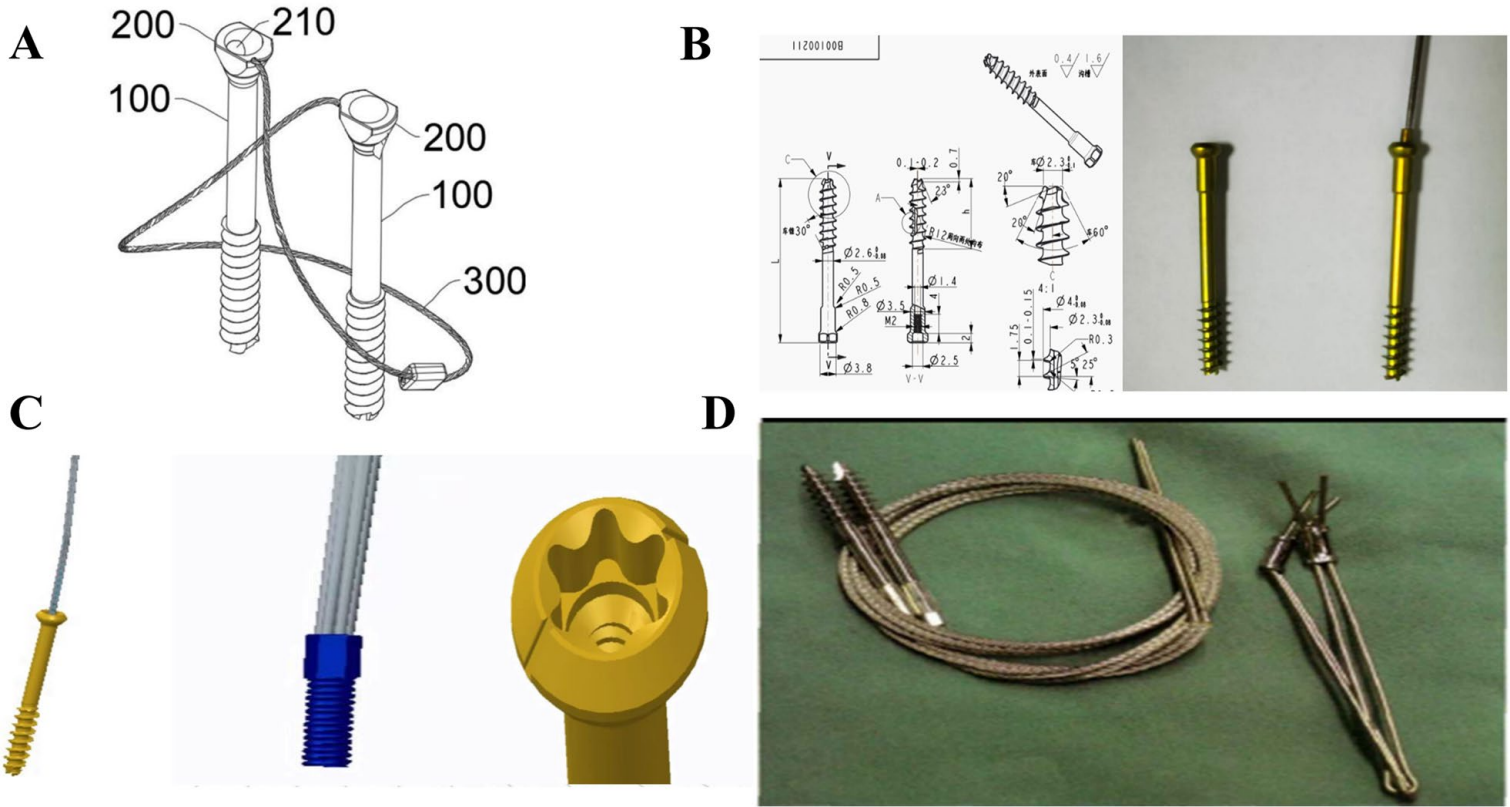
The design sketches and product images of SCIS: **A** **B**: Design sketches; **C**: Simulation image of SCIS; **D** SCIS product

SCIS contains cannulated screws and traction cables (Fig. 1). The head of the screw is equipped with a locking hole (Fig. 1C, 210), which is matched with end of the traction cable (Fig. 1C, 300). In this design, the sharp tip of cannulated screws enables self-tapping, simplifying surgical instrumentation and greatly improving surgical efficiency.

The use of cannulated screws eliminates the need for secondary screw insertion after initial drilling. Based on our experimental testing on cadaveric specimen (unpublished), this approach allows for screw insertion under the guidance and positioning of the presetting *K*-wire. The screw head is threaded to form dual compression with the distal threads when fixing fractures, increasing the fixation strength. The screw and cable have a detachable design: this enables optimal angular positioning of the traction cable for patellar binding and fixation prior to screw fixation of the fracture fragment. Additionally, it prevents excessive entanglement of the traction cable during the insertion of the screw.

### Establishment and analysis of finite element models

#### Establishment of the patella model

First, the patellar solid model was established. A 25-year-old male volunteer (height 180 cm, weight 80 kg) was positioned in a supine position on a CT scan table with the knee joint in a neutral position. A 64-slice spiral CT scanner (Siemens Sensation 64) was used to perform 3D scanning with a slice thickness of 0.5 mm and an interval of 0.8 mm. The acquired two-dimensional image data were saved in DICOM format and imported into the Mimics software(Belgium, v20.0). The appropriate gray scale was adjusted to obtain a clear skeletal contour, which was exported as an STL format file after surface processing. The resulting 3D models in STL format were imported into Geomagic (American, v12.0) software to carry out the reverse engineering reconstruction. The 3D model is segmented, smoothed, polished, denoised and a series of other image processing to enter the polygon processing stage. Then the curved surface sheet distribution operation is performed on the model, and finally the surface is encapsulated and materialized to generate the geometric model of the patella. The final model was saved in IGES format for fracture creation and further analysis.

#### Establishment and categorization of internal fixation models

A simulated transverse fracture, positioned precisely at the midpoint of the patella, was generated (classified as AO/OTA 34-C1) [18]. According to the existing model dimensions, 3D solid modeling was performed using Solidworks to complete the 3 group internal fixator modeling: (1) *K*-wire tension band; (2) SCIS; (3) Cable-pin system. Then the internal fixation models were imported into Workbench software for Boolean calculation.

#### FE analysis

The material parameters of each bone used in the calculation were from previous study [18]. Young's modulus of cortical bone and trabecular bone of the patellar were set to 1000, 207, and 50 MPa, respectively. The Poisson ratios were set to 0.3, 0.3, and 0.1, respectively. Internal fixation instruments (screws, Cable-pin and SCIS) consist mainly of stainless steel, of which Young's modulus and Poisson ratio were set as 200Gpa and 0.3.

Two screws or *K*-wires were positioned in parallel within the central third of the patella, situated either 5 or 10 mm from the anterior patellar surface. Applying anterior tension forces of 400N and 800N above the patella, with traction angles set at 0° and 45°. Contact settings between different components were defined based on real-world conditions: a friction coefficient of 0.2 was applied between metal internal fixations, a friction coefficient of 0.45 was utilized between fracture surfaces, and a friction coefficient of 0.3 was established between internal fixations and the patella [19].

## Results

### *The max. displacement of the fragment and max. gap when screw or k-wire was placed 5 *mm* from the anterior aspect of the patella under the loading of 400&800N in the direction of 0°*

**Fig. 2:**
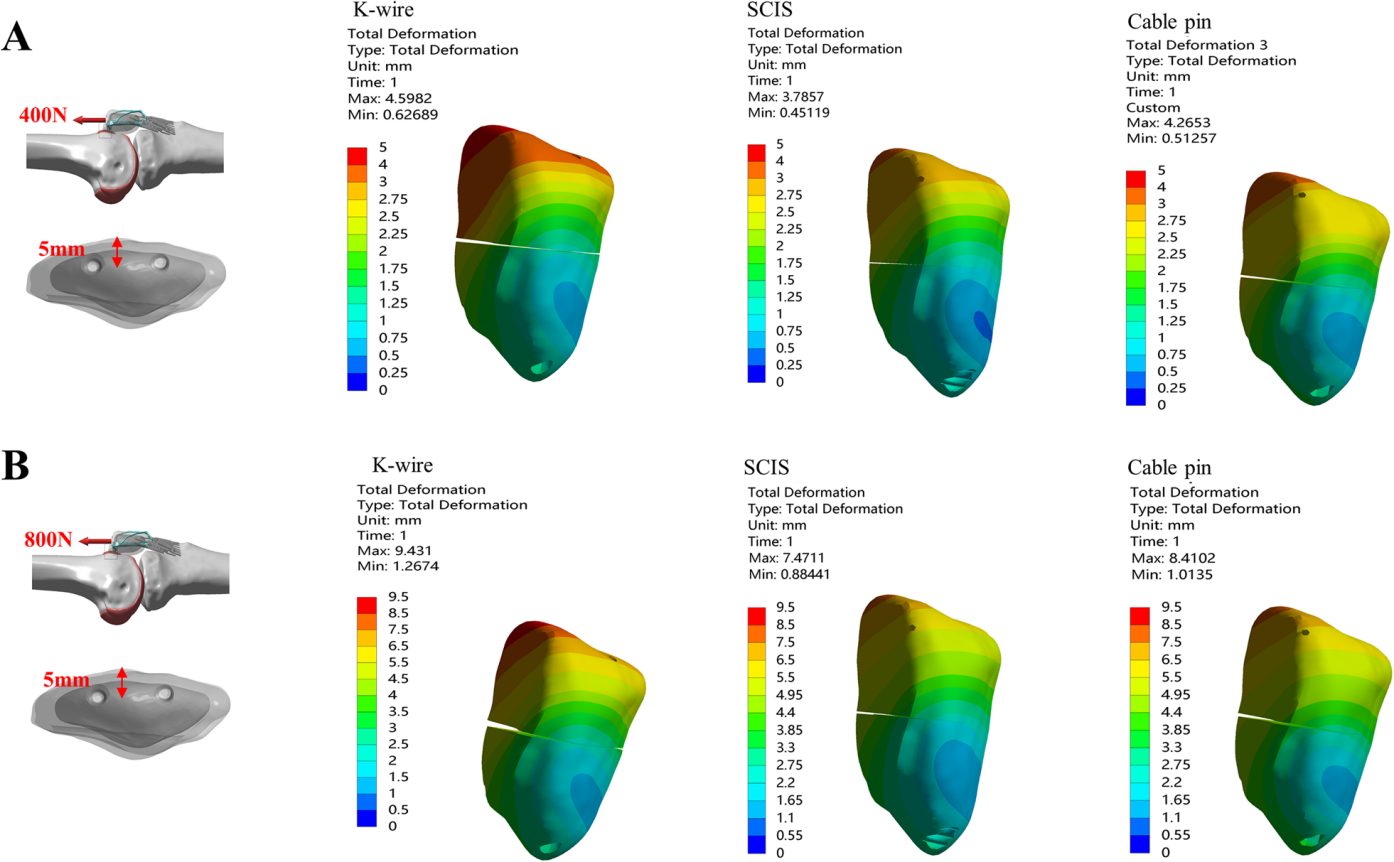
Comparison of the Max. displacement of the fragment and Max. gap between *K*-wire, SCIS and Cable Pin under the condition of 0–5 mm-400N(**A**) and 800N(**B**)

Three fixation methods for treating patella fractures under two loading conditions at the direction of 0° were tested when the *K*-wire /screw was positioned 5 mm anterior to the patella (Fig. 2). The outcome included the maximum displacement of the fragment and the maximum gap opening.

 Under the condition of 400N loading, SCIS had the smallest displacement (3.79 mm) and fracture gap (0.37 mm). Under 800N loading, SCIS had the smallest displacement (7.47 mm) while and the smallest fracture gap (0.62 mm). In summary, the SCIS allowed the most micromotion at the fracture site under both loading conditions tested (Table 1).

**Table 1:** Maximum displacement of the fragment and maximum gap under various conditions

	*K*-wire		SCIS		Cable-pin	
	400N	800N	400N	800N	400N	800N
*Loading direction 0°*						
5 mm depth						
Max. displacement	4.60	9.43	3.79	7.47	4.27	8.41
Max. gap	0.43	0.71	0.37	0.62	0.48	0.68
10 mm depth						
Max. displacement	3.76	7.33	2.95	5.91	3.19	6.21
Max. gap	0.42	0.70	0.14	0.27	0.27	0.42
*Loading direction 45°*						
5 mm depth						
Max. displacement	0.43	0.72	0.22	0.42	0.27	0.50
Max. gap	0.30	0.48	0.05	0.10	0.14	0.22
10 mm depth						
Max. displacement	0.42	0.76	0.30	0.58	0.38	0.68
Max. gap	0.30	0.50	0.18	0.34	0.26	0.43

### *The max. displacement of the fragment and max. gap opening when the screw or K-wire was placed 10 *mm* from the anterior aspect of the patella under 400&800N in the direction of 0°*

**Fig. 3:**
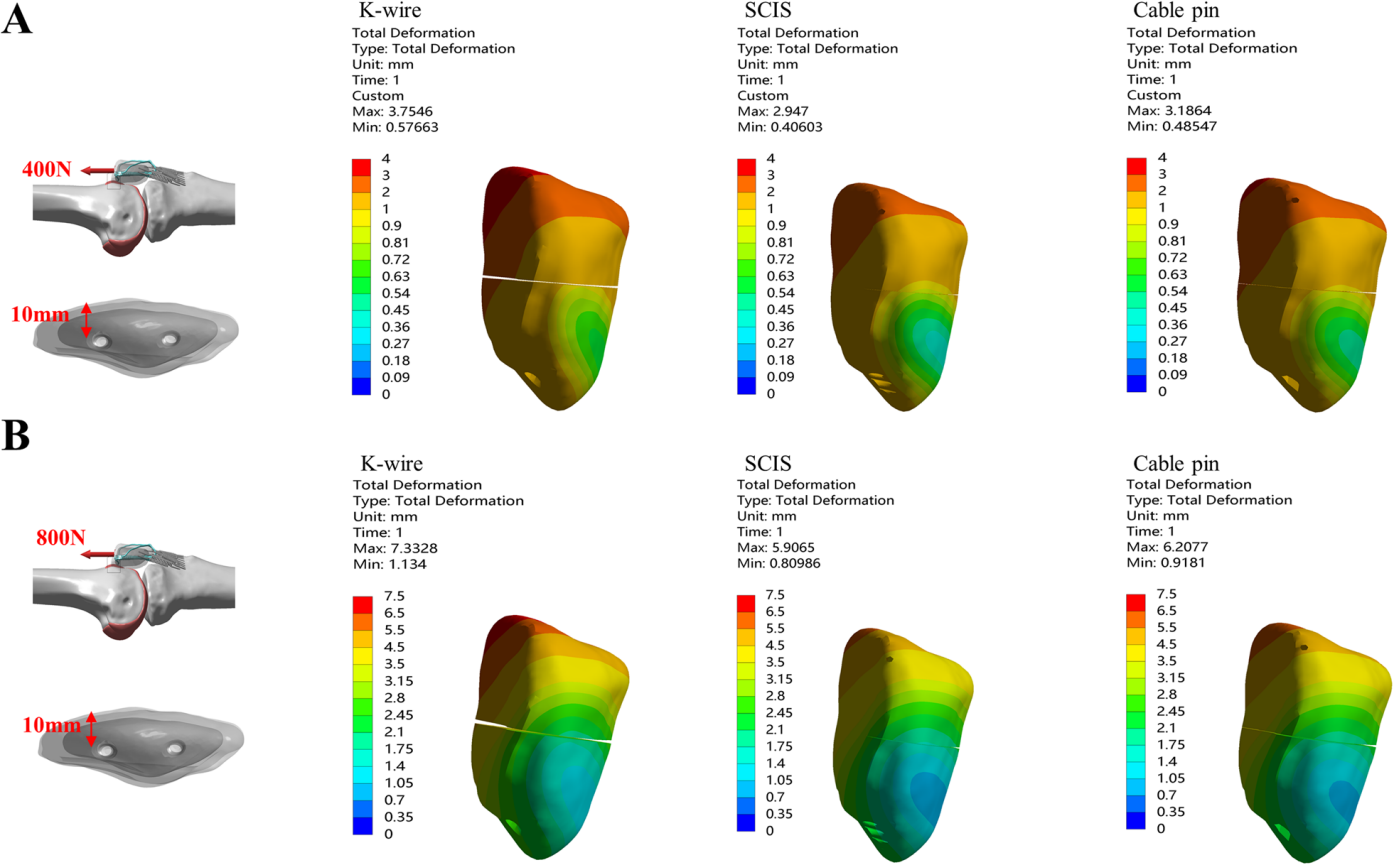
Comparison of the Max. displacement of the fragment and Max. gap between *K*-wire, SCIS and Cable Pin under the condition of 0–10 mm-400N(**A**) and 800N(**B**)

Three fixation methods for treating patella fractures under two loading conditions at the direction of 0° were tested when the *K*-wire /screw was positioned 10 mm anterior to the patella (Fig. 3).

Under the condition of 400N loading, SCIS had the smallest patella displacement (2.95 mm) and fracture gap (0.14 mm). Under the condition of 800N loading, Cable-pin had the smallest patella displacement (6.22 mm) and SCIS had the smallest fracture gap (0.27 mm, Table 1).

### *The max. displacement of the fragment and max. gap opening when the screw or K-wire was placed 5 *mm* from the anterior aspect of the patella under 400&800N in the direction of 45°*

**Fig. 4:**
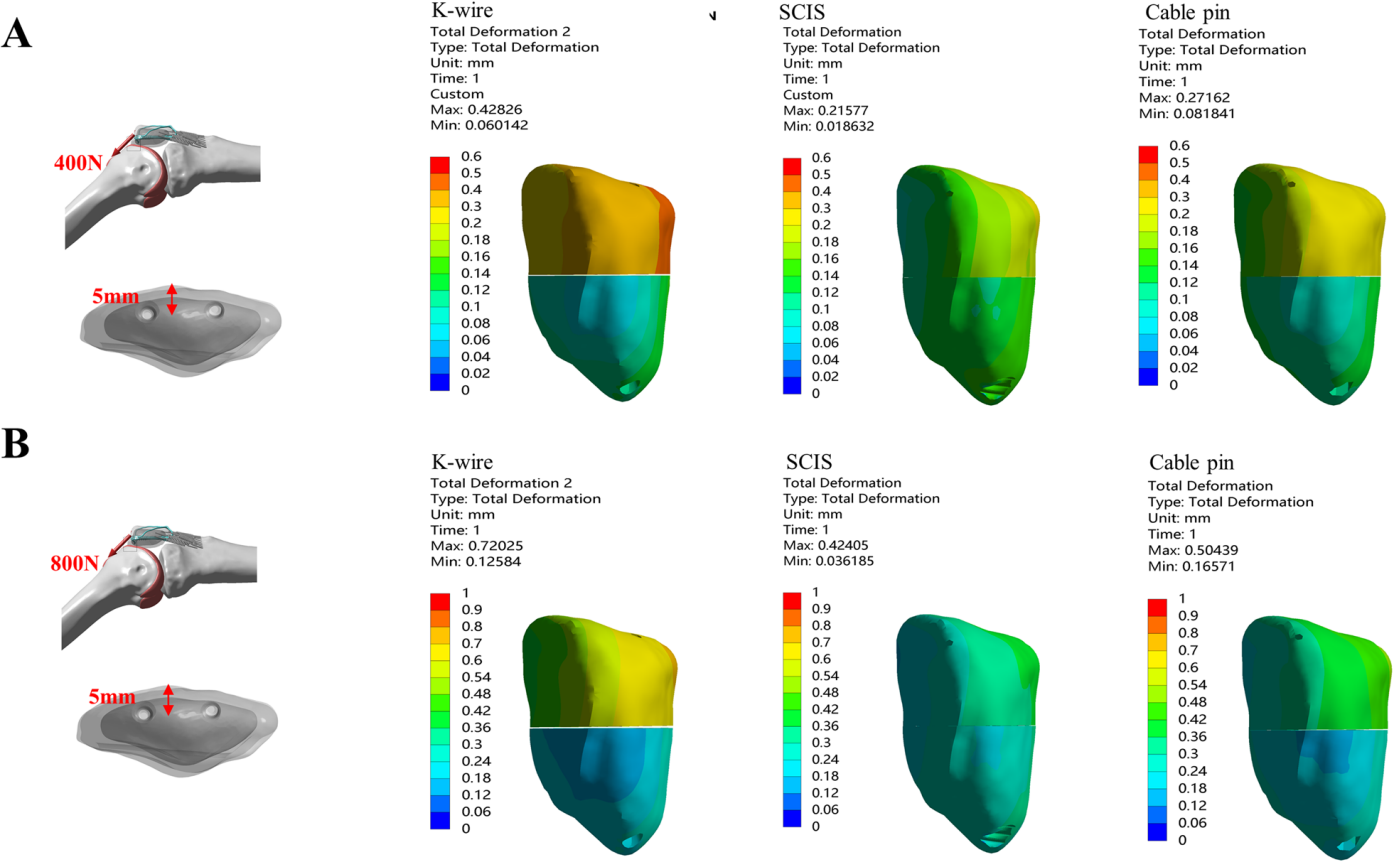
Comparison of the Max. displacement of the fragment and Max. gap between *K*-wire, SCIS and Cable Pin under the condition of 45–5 mm-400N(**A**) and 800N(**B**)

We compared three fixation methods for patella fractures under two loading conditions (400N and 800N) at the direction of 45°when screw or *K*-wire was placed 5 mm from the anterior aspect of the patella (Fig. 4).

Under 400N loading, *K*-wire band had the larges displacement (0.43 mm) and fracture gap (0.30 mm). SCIS had the smallest displacement (0.22 mm) and fracture gap (0.05 mm). Under 45°-800N loading, the *K*-wire band had the largest displacement (0.72 mm) and fracture gap (0.48 mm). SCIS had the smallest displacement (0.42 mm) and fracture gap (0.10 mm, Table 1).

Under 400 N loading in the direction 45°, SCIS with screw placing at 5-mm depth reduced the maximum fragment displacement by 49.62% and 26%, respectively, compared with the *K*-wire and Cable-pin group. Meanwhile, the gap opening in SCIS was reduced by 83% and 59.8% compared with the *K*-wire and Cable-pin group.

### *The max. displacement of the fragment and max. gap opening when the screw or K-wire was placed 10 *mm* from the anterior aspect of the patella under 400&800N in the direction of 45°*

**Fig. 5:**
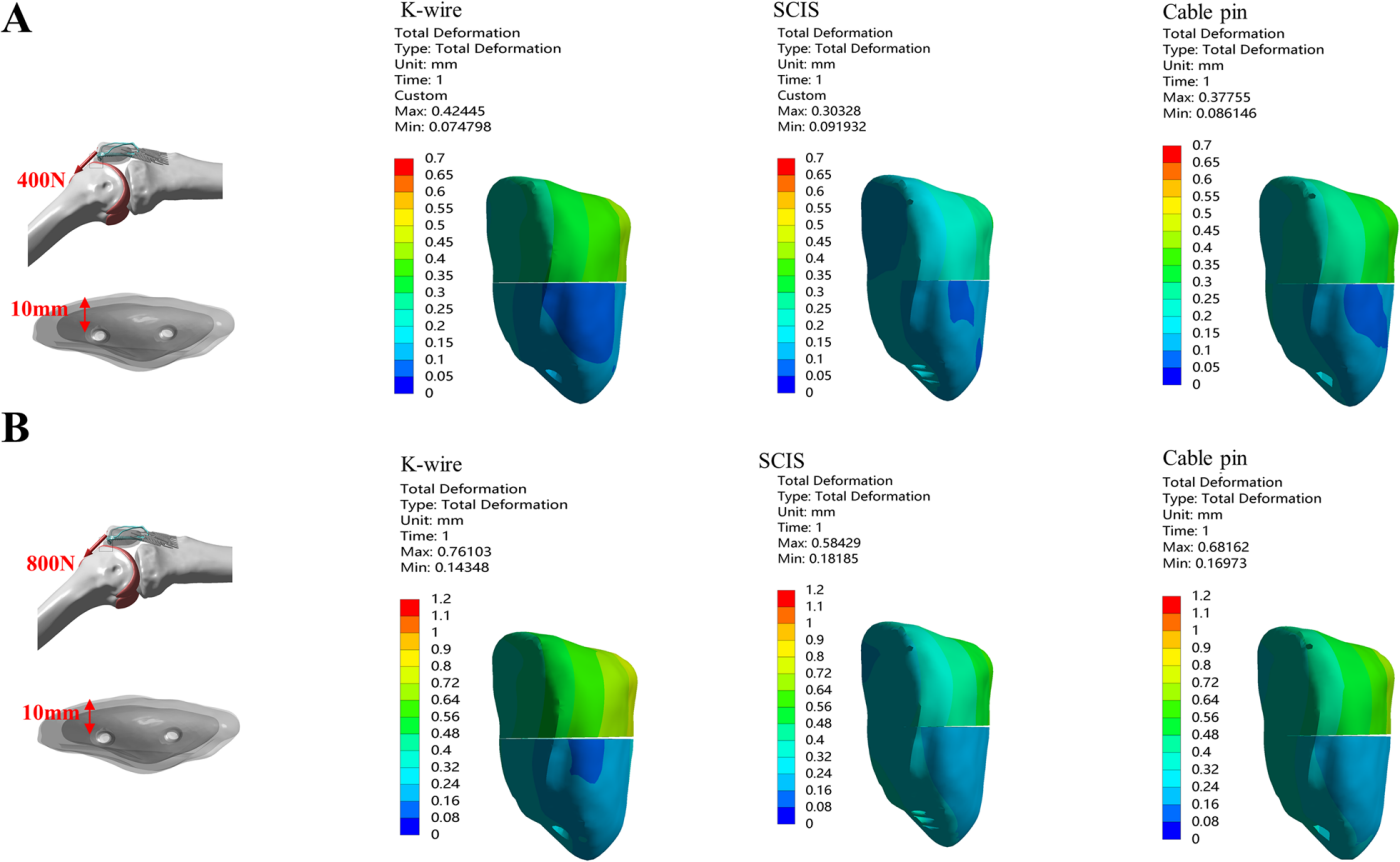
Comparison of the Max. displacement of the fragment and Max. gap between *K*-wire, SCIS and Cable Pin under the condition of 45–10 mm-400N(**A**) and 800N(**B**)

Three fixation methods for treating patella fractures under two loading conditions at the direction of 45° were tested when the *K*-wire /screws were positioned 10 mm anterior to the patella(Fig. 5).

Under the condition of 400N loading, *K*-wire group had the largest Max. displacement (0.42 mm) and Max. gap(0.30 mm). SCIS had the smallest displacement (0.3 mm) and gap (0.18 mm). Cable-pin was intermediate between the two (0.38 and 0.26 mm). Under 800N loading: *K*-wire band again had the largest displacement (0.76 mm) and gap (0.50 mm). SCIS again had the smallest displacement (0.58 mm) and gap (0.34 mm). Cable-pin was intermediate between the two groups (Table 1).

## Discussion

In this study, we conducted a finite element analysis of the novel screw-cable integration system (SCIS) for treating transverse patella fractures. The findings provide valuable insights into the biomechanical performance and potential clinical implications of this novel system. Our results demonstrate that the SCIS effectively provided stability to the fractured patella. The displacement analysis indicated that the system has the potential to improve fracture healing compared to conventional techniques.

The most common strategy for treating transverse patellar fractures is modified tension band wiring (MTBW), which transforms tension force into pressure force [8, 20, 21]. However, Zedric et found that no notable variance in interfragmentary pressures was observed on the articular surface during knee flexion by tension band wire which led them to consider the tension band wire “as a static fixation principle rather than a dynamic one [22]. This revolutionary theory provides the foundation for the development of new minimally invasive fixation instruments, aiming to replace the Kirschner wire in the conventional MTBW technique. Based on this, we creatively proposed an integrated design of SCIS combining the principle of tension band wiring (TBW) principle and the Cable-pin system. The novel system also provided technical support for the minimally invasive treatment of patellar fractures. When designing SCIS, we considered that the following advantages might be integrated into the product: (1) Anti-rotation and dual pressurization effect: The design of a headless compression screw generates dual compression along the entire deforming fracture plane for superior stabilization; (2) Reduced stimulation: the design of a headless screw can reduce soft tissue irritation, lower the risk of infection, and promote early mobility. (3) Integrated screw-cable locking: this prevents screw back-out and cable loosening. The two components reinforce each other through interlocked compression for enhanced stability. (4) Amenable to minimally invasive surgery: the technique is straightforward surgically—percutaneous fracture reduction is followed by cannulated screw insertion. Once a satisfactory position is achieved, the screw is directly implanted. The wire can be tightened with a tensioner before or after screw locking based on fracture pattern. The approach can be tailored for various fracture types and its certainty still needs to be verified.

In the present study, we found that SCIS reduced the max displacement by 12.65%(0–400N), 20.78%(0–800N), 49.62%(45–400N) and 41.12%(45–800N) when the fixation was placed at the depth of 5 mm compared to TBW with *K*-wire. The results demonstrated benefits in relation to interfragmentary motions at the fracture site, which was similar to the conclusion of a series of other studies [13, 23, 24]. This might be caused by the following reasons. First, the interfragmentary compression provided by cannulated screw fixation creates a more rigid construct compared to the flexible fixation of *K*-wires. Nikhil Drolia1et al reported fixing closed transverse patella fractures with two 4.5 mm cannulated screws enables quicker fracture union, improved knee range of motion, and fewer hardware-related complications issues compared to using Kirschner wires [11]. Second, screw threads engage the bone for solid purchase versus smooth *K*-wires which rely solely on geometric configuration for stability. Rigid fixed-angle screw fixation better restores patellar biomechanics and resists displacement forces during knee motion. Backing out of implants and loss of reduction is less likely with buried screws versus percutaneous protruding *K*-wires.

The SCIS also presents advantages compared to the Cable-pin system. Our system uses headless compression screws to allow bi-cortical purchase, which was inspired by the research of Chen et al. [18]. Their study demonstrates that, in comparison to the partially threaded screws, headless compression screws exhibit better grip strength and provide greater stability in fracture fixation. The threads at both ends of the screw achieve compression along the entire deforming fracture plane for superior stabilization versus Cable-pin which uses partially threaded screws and rely on focal compression. This dual compression mechanism provides superior fixation, which was demonstrated by our results. In addition, SCIS design preserves bone stock as guiding wires can remain in place during screw insertion, without the need for repeatedly drilling out and reinserting them at different angles, avoiding unnecessary bone loss. The design of the headless screw avoided anterior knee pain from prominent screw heads irritating overlying soft tissues and allowed for early flexion and extension movements.

The placement position of screws or Kirschner wires relative to the anterior surface of the patella has always been a subject of controversy [25]. In our study, when the force direction is at 0°, regardless of whether the load is 400N or 800N, placing the SCIS at 10 mm results in the smallest fracture fragment displacement and gap opening. In contrast, when the force direction is at 45°(flexion), placing the SCIS at 5 mm results in the smallest fracture fragment displacement and gap opening. This partially aligns with the findings from Chang's study. According to Chang’s study, placing screws close to the surface (5 mm depth from anterior surface) proved more effective in minimizing fracture gap opening, especially during knee flexion, while deeper screw placement (10 mm depth) demonstrated superior ability in maintaining fracture site contact during full knee extension [18, 26].

A finite element analysis conducted by Ling et al. found that during knee flexion at a 45° angle, positioning *K*-wires closer to the articular surface at the posterior end offers the best stability and stress distribution for the fracture [9]. Their study did not provide specific depth from the anterior patella surface. Further exploration and clinical trials are needed to understand the clinical efficacy of the various screw proximity. Moreover, the precise placement of screws or Kirschner wires during surgery according to preoperative planning is notably challenging, which might be potentially addressed under surgery robotic assistance.

It is important to consider the limitations of our study. First, the realism of the present study is limited as the parameters used in FE modeling did not reach a consensus [27]. FE analysis is just a simulation, which can serve as a reference. Real data validation still requires the use of cadaveric specimens, biomechanical studies, and even clinical experiments. Second, bone healing is one of the key factors to consider when designing a novel fixation instrument. The effect of screw configurations on bone healing was not considered in the present study, which was proposed by the study of Travascio et al. [28]. Third, the stretching of the infrapatellar nerve by the novel instrumentation is a factor that must be taken into account [29]. However, FE analysis is unable to directly detect the effect of SCIS on nerve stretching, which is a limitation of this experiment. Last, the range of loads used in FE(400N/800N) does not encompass all loading situations after patellar fracture surgery. It is worth noting that the chosen load exceeds the failure loads reported by Massey et al. in their study on patellar tendon repair [30]. Thus, the SCIS applied in our study can be considered safe for similar postoperative treatment scenarios.

## Conclusion

The novel screw-cable integrated system (SCIS) demonstrated improved biomechanical stability for transverse patellar fractures compared to modified tension band wiring with Kirschner wires and the Cable-Pin system. Finite element analysis showed SCIS substantially reduced fracture fragment displacement and gap opening under various loading conditions. In future, SCIS holds the potential to be combined with the use of orthopedic robots to achieve more precise minimally invasive surgeries. SCIS may also find application in clinical treatments such as olecranal fractures and comminuted patella fracture. Real data validation still requires the use of cadaveric specimens, biomechanical studies, and even clinical experiments.

## Data Availability

All data generated or analyzed during this study are included in this published article.

## Abbreviations

FE: Finite element
MTBW: Modified tension band wiring
SCIS: Screw-cable integrated system
